# Effect of Ru, Rh, Mo, and Pd Adsorption on the Electronic and Optical Properties of Anatase TiO_2_(101): A DFT Investigation

**DOI:** 10.3390/ma12050814

**Published:** 2019-03-10

**Authors:** Peng Gao, Libin Yang, Songtao Xiao, Lingyu Wang, Wei Guo, Jinghao Lu

**Affiliations:** 1 College of Chemical Engineering and Materials Science, Tianjin Key Laboratory of Brine Chemical Engineering and Resource Ecological Utilization, Tianjin University of Science and Technology, Tianjin 300457, China; 15822843435@163.com (P.G.); 18822057890@163.com (W.G.); L17600886312@163.com (J.L.); 2 Department of Radiochemistry, China Institute of Atomic Energy, Beijing 102413, China; xiao200112@163.com (S.X.); 18810128827@163.com (L.W.)

**Keywords:** density functional theory (DFT), anatase TiO_2_, TiO_2_(101) plane, electronic structure, optical absorption, adsorption mechanism

## Abstract

Adsorbed metal atoms and metal doping onto TiO_2_ can effectively enhance the optical and photocatalytic activity of photocatalytic efficiency of titanium dioxide (TiO_2_), favoring the extension of its optical absorption spectrum and the efficiency of hydrogen generation. To investigate the possible mechanism causing potential improvement of photocatalytic activity, the electronic and optical properties of the anatase TiO_2_(101) plane with different adsorbed metal atom have been theoretically calculated through density functional theory (DFT) method. Adsorption of Pd and Ru atoms increases the delocalization of the density of states, with an impurity state near the Fermi level. Moreover, the investigated adsorbed metal atoms (Mo, Pd, Ru, Rh) narrow the band gap of anatase TiO_2_, thus enhancing the probability of photoactivation by visible light. The orbital hybridization of the d orbit from the adsorbed metal atom and the p orbit from the O of the defect site increases the Schottky barrier of the electronic structure.

## 1. Introduction

Concerning global energy and environmental issues, the development of technologies for environmental pollution control and clean and efficient energy is very important. Beginning with the pioneering research on photocatalysis water splitting of titanium dioxide (TiO_2_) electrodes reported in 1972 [1], photocatalysis technology using semiconductors and solar energy has been attracting tremendous attention [2], and various particulate overall water splitting photocatalysts have been investigated [3,4,5,6]. Titanium dioxide (TiO_2_) has been widely applied in many fields, including pigments productions, photocatalysis, hydrogen storage, and production and novel solar cells, thanks to its favorable properties including nontoxicity, high stability, abundant availability, etc. [7,8]. However, as a wide band-gap semiconductor (3.2 eV for anatase and 3.0 eV for rutile), TiO_2_ can only absorb ultraviolet (UV) radiation, which amounts to about 5% of the solar energy [9]. To overcome these shortcomings, it is critical to reduce the band gap of semiconductors, such as TiO_2_, so that the absorption properties might match well with the solar spectra. In order to achieve this target, numerous attempts have been made with respect to modifying the energy band and structure of TiO_2_, such as ion doping or metal adsorption, semiconductor coupling, dye sensitization, etc. [10,11,12,13,14]. Compared with the others, metal adsorption or ion doping is the most direct and efficacious method.

Among the reported natural polymorphs of TiO_2_ (rutile, anatase, and brookite), anatase phase commonly exists in TiO_2_ nano-scale materials [15,16]. Under equilibrium conditions, both the natural and synthetic anatase TiO_2_ crystals grow along (001) direction, which exposes the low-energy (101) facets thereby minimizing the surface energy and increasing the crystal stability by forming truncated bipyramidal shapes [17,18]. Moreover, theoretical calculation reveals that the surface energy of major exposed facets of anatase TiO_2_ crystal follows the order (110) > (001) > (100) > (101) [19,20,21]. Thus, the (101) plane is the most stable one in anatase TiO_2_ and represents the dominant crystalline face [22].

Adsorption of metals onto TiO_2_ are an effective method to narrow the band gap of TiO_2_ and improve the photocatalytic activity [23]. Recently, theoretical calculations have been widely used to study the photocatalytic principle of metal being doped into the inner lattice of TiO_2_ [24]. The results of the theoretical calculations demonstrated the mechanism of the photocatalytic activity intensified by the doped metal cluster. Furthermore, some studies on the deposition of metal onto the surface of TiO_2_ are mainly focused on the analysis of photocatalytic activity from the experimental point of view [25,26,27]. However, fewer researchers have focused on the changes of surface electronic structure caused by the deposition of metal clusters. Furthermore, the Mulliken charges, electrostatic potential, and averaged differences were investigated to provide evidence for practical applications. It is critical to explain at the atomic scale the mechanism of metals being adsorbed on the surface of TiO_2_ to improve photocatalytic activity by researching the electronic structure and optical properties of metal cluster deposited TiO_2_.

In this work, the electronic and optical properties of structures composed of metals (M = Ru_n_, Rh_n_, Mo_n,_ and Pd_n_, *n* = 1−2) adsorbed on the anatase TiO_2_ (101) plane (hereafter abbreviated as M/TiO_2_) are investigated by density functional theory (DFT) calculations to indicate the mechanism for narrowing band gap and intensifying the migration of photo-induced carriers.

## 2. Computational Methods and Models

All calculations were based on density functional theory (DFT) with the exchange-correlation functional at the generalized gradient approximation (GGA) level parametrized by Perdew, Burke, and Ernzerh (PBE) [28], as implemented in the Cambridge Serial Total Energy Package (CASTEP) codes [16], combined with ultrasoft pseudopotentials (USPP) [29]. In this procedure, the electronic wave function was expanded in the plane wave basis sets with a cutoff energy of 400 eV. The minimization algorithm was the Broyden-Fletcher-Goldfarb-Shanno (BFGS) scheme [30]. The k-points grid sample of the Monkhorst Pack scheme was set as 3 × 2 × 2 in the irreducible Brillouin zone for the slab model of M/TiO_2_. Moreover, the atomic coordinates were optimized by minimizing the total energy and atomic forces to obtain accurate results. The convergence criteria were set as follows: the maximal force on the atoms was 0.01 eV/Å, the stress on the atomic nuclei was less than 0.05 GPa, the maximal atomic displacement was 2 × 10^−3^Å, and the maximal energy change per atom was 2 × 10^−5^ eV.

**Figure 1:**
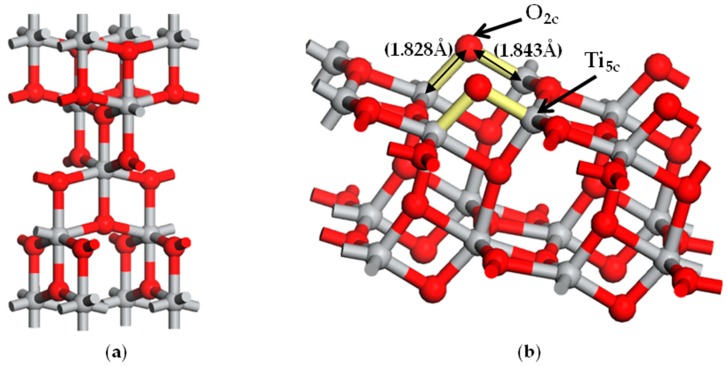
Model of the adsorbed structure: (**a**) the unit cell of anatase TiO_2_; and (**b**) the slab of the anatase TiO_2_(101) plane, the distances represented in Figure 1b are the calculated lengths of the O_2c_-Ti_5c_ bond (1.843 Å) and O_2c_-Ti_6c_ bond (1.828 Å). Gray and red spheres represent Ti and O atoms, respectively.

The unit cell of anatase TiO_2_ (as shown in Figure 1a) has a symmetry of space group *D*^19^_4h_-*I*_41_/*amd* [31], the lattice constants of the primitive anatase TiO_2_ from the literature and the relative errors of GGA calculations are listed in Table 1 [31]. A slightly larger relative error of c was still less than 2.5%, so the computational methods are reasonable. The calculated band gap of anatase phase TiO_2_ is 2.0 eV. This is less than the experimental value of 3.2 eV. The density functional theory omits the discontinuity of the electron exchange correlation potential, which results in a deviation of 30–50% of the basic band gap width from the experimental value. The scissors operator effectively describes the difference between the theoretical and experimental band gap values. In order to compensate for the band gap difference, the scissor operator is set to 1.2 eV during the analysis. Fortunately, the above contents being omitted has not affected the computational results of the electronic structure. The (101) anatase was simulated by periodic (2 × 2) slab models with four O–Ti–O layers and a 10 Å vacuum layer. A single slab is shown in Figure 1b. For the optimization of the unit cell geometry, the bottom two O–Ti–O atomic layers were considered as fixed and the upper two atom layers were relaxed.

The valence electron configurations Ti (3d^2^4s^2^), O (2s^2^2p^4^) were analyzed for their contribution to the photocatalytic activity. The bonding of anatase TiO_2_ between anions and cations as a tetragonal system shows mixed bonding characteristics: dominant ionic binding and partial covalent bonding [32]. The coordination numbers for anions and cations are 6 and 3, respectively. The octahedral phase of [TiO_6_]^8−^ is interconnected by common bonds to form an anatase. Compared with the bulk phase, the coordination environment of anions and cations on the top of the layer with a clean anatase with (101) face is changed: two-fold coordinated anions and five-fold coordinated cations are present on the first and second atomic layers, respectively (Figure 1b). Furthermore, on the anatase TiO_2_(101) plane, the chains of two-fold coordinated O atoms called “bridging” are parallel to the (101) direction and rise above the plane of the surface. We investigated the adsorption of noble metal M (M = Ru_n_, Rh_n_, Mo_n,_ and Pd_n_, *n* = 1−2) on the sites of two-fold coordinated anions (O_2c_, in other words, bridging O atoms) and five-fold coordinated cations (Ti_5c_) in the anatase TiO_2_(101) plane.

The stability of the electronic structures of M/TiO_2_ (101) had been estimated by the adsorption energy E_ads_ = E_atom_ + E_surface_ − E_atom/surface_. Here, E_atom_ is the energy of an isolated metal atoms, E_surface_ is the energy of clean (101) surface slab, and E_atom/surface_ is the total energy of the surface with metal atom (M) adsorption [33].

## 3. Results and Discussion

**Figure 2:**
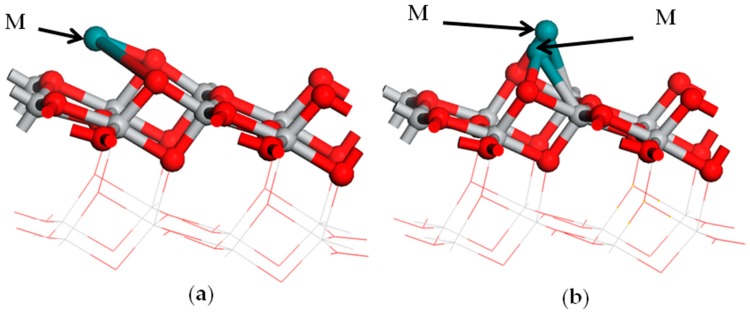
The structure model of the metal adsorption on the TiO_2_ surface: (**a**) the monoatomic adsorption structure; (**b**) the diatomic adsorption structure. Medium shade of blue shadow, gray, and red spheres represent M of metal, Ti, and O atoms, respectively.

Two configurations of metal M atoms’ adsorption on the anatase TiO_2_(101) plane were modeled. The optimized adsorption configurations are given in Figure 2. In the monoatomic adsorption configuration, one of the M atoms was adsorbed between two O_2c_ atoms on the anatase TiO_2_(101) plane and formed two M-O bonds (shown in Figure 2a). Another was in the diatomic configuration, and double M atoms were bonded between two O_2c_ and Ti_5c_ atoms on the anatase TiO_2_(101) plane (shown in Figure 2b).

### 3.1. Structure of Surface Adsorption

The calculated O-Ti bond lengths on free (101) anatase surface (i.e., pristine anatase) were 1.843 Å and 1.828 Å (as indicated in Figure 1b) for O_2c_-Ti_5c_ and O_2c_-Ti_6c_, respectively. Hence, the average O-Ti bond length for pristine (101) surface was found to be 1.8355 Å. The presence of an adsorbed metal atom (M) modifies the electronic structure and the bond configuration: a summary of the structural parameters modified by Ru, Rh, Mo and Pd adsorbed atoms is reported in Table 2 for both monoatomic and diatomic adsorption structure. More in detail, Table 2 reports the calculated average O-Ti bond length, the difference between O-Ti bond length of free and metal-adsorbed (101) anatase surface, the adsorption energy, the electrostatic potential and the Mulliken charge (see next for further description).

In diatomic configuration, the (101) plane is significantly relaxed and O_2c_ atoms are dragged out from their primitive position due to the adsorption of metal atoms. The bonds of O_2c_-Ti_5c_ and O_2c_-Ti_6c_ were elongated. Compared with the primitive anatase TiO_2_ (101) plane, in both adsorption configuration, the largest and smaller increase for O_2c_-Ti_5c_ and O_2c_-Ti_6c_ bond lengths are obtained via Mo adsorption and Ru adsorption, respectively. Compared with the diatomic adsorption configuration, the O_2c_-Ti_5c_ and O_2c_-Ti_6c_ bond length change is smaller than the monoatomic configuration. Furthermore, the changes in O_2c_-Ti_5c_ and O_2c_-Ti_6c_ lengths affect the stability of surface adsorption, as it is more stable for smaller bond changes.

The adsorption-induced shifts of average O_2c_-Ti_5c_ and O_2c_-Ti_6c_ bond lengths in the two investigated adsorption configurations indicate that the adsorption of metal atoms on the anatase TiO_2_(101) plane is chemisorption, resulting in a change in the local O-Ti bond (indicated as Δ in Table 2). Table 2 also reports the calculated adsorption energies for the M atoms: it is noted that all of them are negative. This indicates a favorable process [34]; i.e., the investigated metal should be steadily adsorbed onto the anatase TiO_2_(101) plane.

### 3.2. Electronic Structure

The potential distribution can provide information about the electric field and transfer of photogenerated charges in the M/TiO_2_ (101) systems. As reported in Table 2, the average electrostatic potential of Ru/TiO_2_ (101) in the monoatomic structures (4.23 eV) was lower than that of the free TiO_2_(101) surface (7.033 eV).

In the monoatomic adsorption configuration, the final built-in electric field of Ru/TiO_2_(101) pointed from the metal Ru layer to the TiO_2_(101) surface layer. Because of the differences of the average electrostatic potential between the primitive TiO_2_ and the monoatomic adsorption configuration Ru/TiO_2_(101), the mobile (i.e., photogenerated) electrons will tend to localize close to the adsorbed Ru atom, thus leading to separation of the photogenerated carriers [32]. Compared with the monoatomic adsorption configuration. Four metals atoms adsorbed in the diatomic configuration have a smaller value of the average electrostatic potential. This was favorable for the capture of photogenerated carriers. However, the difference between the average electrostatic potentials was different. For Ru and Pd atoms adsorbed in the two configuration have smaller value of difference between the average electrostatic potentials (the value of Ru adsorbed was 2.803, 2.893 eV), this was favorable for the capture of photogenerated carriers. However, Rh and Mo atoms adsorbed in the two configuration have bigger value of difference between the average electrostatic potentials (the value of Rh adsorbed was 6.1, 6.15 eV).

To reveal the separated mechanism of the photogenerated carriers, the charge distribution of M/TiO_2_(101) such as Mulliken charge analysis proved to be very advantageous. The data of Mulliken charge analysis are shown in Table 2. From charge transfer at the M/TiO_2_(101), the amount of monoatomic Rh/TiO_2_(101) surface electron transfer in the structure is the largest on these M/TiO_2_(101) surface, followed by the monoatomic Ru/TiO_2_(101) surface. Combined with the results of the average electrostatic potential and the Mulliken charge distribution, electron aggregation occurs at the side of metal-M, while the anatase TiO_2_(101) plane shows electron depletion after M/TiO_2_(101) surface structure formation.

**Figure 3:**
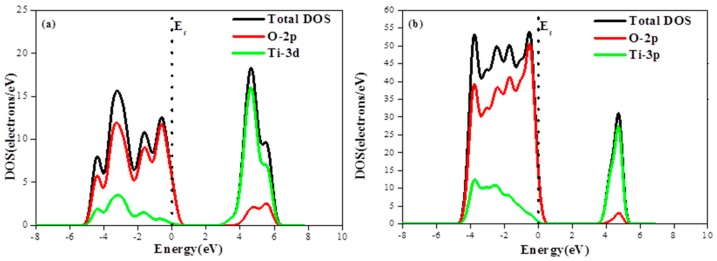
(**a**) Density of states of primitive TiO_2_ and partial DOS of O, Ti atom; and (**b**) the density of states of the anatase TiO_2_(101) plane and partial DOS of O, Ti atom.

The photocatalytic performance can be traced to the surface electronic structure. As shown in Figure 3a, the valence band (VB) and conduction band (CB) of anatase TiO_2_ were predominantly composed of O-2p states and Ti-3d states, respectively.

According to density of states (DOS) calculations, the unrelaxed TiO_2_ exhibits continuous conduction and valence bands extending in energy ranges from 1 eV to 7 eV and from −5 eV to 1 eV, respectively. Regarding the relaxed (101) anatase, its maximum conduction band DOS is found to be about 30 electrons/eV, i.e., 1.5 times higher than that of unrelaxed anatase while its maximum valence band DOS is about four times greater than the unrelaxed anatase one, as shown in Figure 3b.

**Figure 4:**
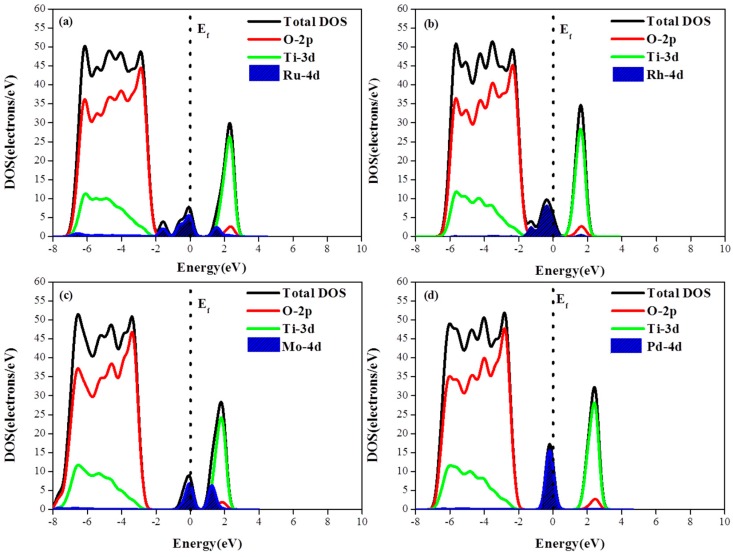

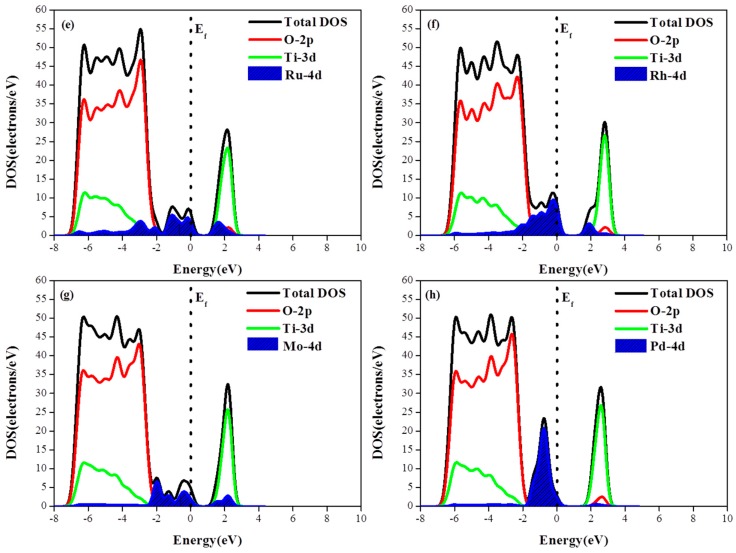
(**a**–**d**) Density of states of M (Pd, Ru, Mo, Rh)/TiO_2_(101) with the monoatomic structure; and (**e**–**h**) the density of states of M (Pd, Ru, Mo, Rh)/TiO_2_(101) with the diatomic structure.

To investigate the photogenerated electronic characteristics of metal adsorbed photocatalysts, the total and partial DOS of the M/TiO_2_(101) systems are shown in Figure 4. Although the adsorbed metal atoms do not change the properties of the primitive TiO_2_, the results of the electronic structure calculation indicates that the metal 4d orbitals contribute to energy levels positioned very close to the Fermi level. Among the various adsorbed metals, Ru exhibits the largest energy dispersion while Pd shows the largest DOS and the energy localization (i.e., smallest energy dispersion) Compared with the monoatomic adsorption structure, the DOS of M-4d in the diatomic adsorption configuration exhibited a larger degree of energy dispersion, i.e., the second adsorbed atom enhanced the DOS energy spread with respect to the previous case. Due to the appearance of M-4d electronic impurity states, a new degenerate energy levels are formed, narrowing the band gap and eventually increasing the probability of TiO_2_ activation via the optical promotion of electrons from the top of the M-4d impurity state to the conduction band.

### 3.3. Optical Absorption

The optical absorption coefficient is related to the complex dielectric function *ε* = *ε*_1_ + і*ε*_2_ by the following equation:

(1) $$\alpha \left(\omega \right)=\frac{2\omega }{c}{\left\{\frac{1}{2}{\left[\varepsilon_{1}^{2}\left(\omega \right)+\varepsilon_{2}^{2}\left(\omega \right)\right]}^{1/2}-\varepsilon_{1}\left(\omega \right)\right\}}^{1/2}$$

Here, we calculated the imaginary part (*ε*_2_) and the real part (*ε*_1_) of the dielectric function via DFT and then the absorption coefficient using Equation (1).

**Figure 5:**
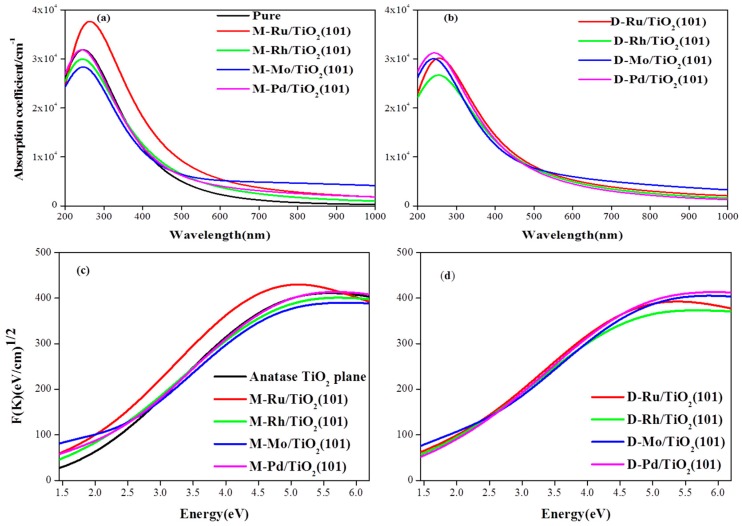
The optical absorption spectra of different adsorption structures: (**a**) the anatase TiO_2_(101) plane and M/TiO_2_(101) with the monoatomic adsorption structures; (**b**) the M/TiO_2_(101) with the diatomic adsorption structure; (**c**,**d**) the Tauc plots of M/TiO_2_(101) with the monoatomic and diatomic adsorption structures, respectively.

The optical absorption spectra in the UV–VIS region were shown in Figure 5. From the Figure 5a,b, the metal adsorbed on the surface of the TiO_2_(101) not only intensified the absorption in the UV region, but also extended to the visible region. In the visible light region, the Ru, Pd/TiO_2_ exhibited a stronger light absorption property than the Mo, Rh/TiO_2_, and the valence band red shift of the Ru/TiO_2_(101) surface was the largest. The relatively strong infrared light absorption of Mo/TiO_2_ could induce the surface plasmonic effects, if the metal Mo are present nanoparticle on the surface of TiO_2_. As is shown in Figure 5c,d, it is found that anatase TiO_2_ plane only responds to UV light and shows smaller absorption activity to the visible light. Generally, metal adsorption or ion doping promotes the shift of the fundamental absorption edges toward the visible light region with a noticeable red-shift effect and the absorption efficiency is larger than anatase TiO_2_ plane. It can be seen that Ru and Pd atoms adsorbed on the surface intensified the absorption and promotes the shift of absorption in the visible light region, as shown in Figure 5c,d.

## 4. Conclusions

Electron aggregation and delocalization occur at the side of the adsorbed metal atom (Ru, Rh, Mo, or Pd) on the anatase TiO_2_(101) plane, and the appearance of (Ru, Rh, Mo, or Pd)-4d electronic impurity states increases the probability of migration of the electrons from the top of the impurity state to the conduction band, as a result of the narrowed band gap.

## Figures and Tables

**Table 1:** Lattice constants for anatase TiO_2_ and their differences with respect to experimental measurements.

Lattice Constants (A)		
Category	a/b	c
This work	3.796	9.712
Experimental	3.782	9.502
Difference	0.368%	2.162%

**Table 2:** Average O_2c_-Ti_5c_ and O_2c_-Ti_6c_ bond lengths (Å) adsorption-induced shift of average bond lengths (in Angstrom units), average electrostatic potentials and the Mulliken charges for the various M/(101)TiO_2_ systems investigated.

Configurations		Average O-Ti Bond Length (Å)	Δ(Å)	E_ads_(eV)	Electrostatic Potential (eV)	Mulliken Charge (e)
Free (101) Anatase		1.8355	0	-	7.033	2.48
Monoat.	Ru	1.8595	0.046	−0.81	4.23	2.23
	Rh	1.8865	0.100	−0.24	0.94	2.4
	Mo	1.9175	0.142	−0.11	1.03	2.45
	Pd	1.8545	0.063	−0.63	3.94	2.30
Diatom.	Ru	1.8815	0.015	−1.04	4.14	2.12
	Rh	1.9357	0.051	−0.52	0.88	2.3
	Mo	1.978	0.082	−0.47	0.66	2.43
	Pd	1.8985	0.019	−0.76	3.81	2.17
